# Low lean mass with obesity in older adults with hypertension: prevalence and association with mortality rate

**DOI:** 10.1186/s12877-023-04326-x

**Published:** 2023-10-03

**Authors:** Qiang Qu, Qixin Guo, Jinyu Sun, Xinyi Lu, Iokfai Cheang, Xu Zhu, Wenming Yao, Xinli Li, Haifeng Zhang, Yanli Zhou, Shengen Liao, Rongrong Gao

**Affiliations:** 1 Department of Cardiology, The First Affiliated Hospital of Nanjing Medical University, 300 Guangzhou Road, Nanjing, 210029 China; 2 Department of Cardiology, Suzhou Municipal Hospital, Gusu School, The Affiliated Suzhou Hospital of Nanjing Medical University, Nanjing Medical University, 26 Daoqian Street, Suzhou, 215002 China; 3 Department of Cardiology, Jiangsu Province Hospital, 300 Guangzhou Road, Nanjing, 210029 China

**Keywords:** Prospective study, Older adults, Sarcopenia, Low lean mass, Appendicular lean mass index, Obesity, Sarcopenic obesity, Low lean mass with obesity, Prevalence, All-cause mortality

## Abstract

**Background:**

The influence of sarcopenic obesity (SO) on overall survival in older adults with hypertension has not been addressed. The aim of this study was to investigate the prevalence and mortality predictive value of various body composition phenotypes, focusing mainly on SO, in older adults with hypertension.

**Methods:**

We included 1105 hypertensive patients aged ≥ 60 years from the National Health and Nutrition Examination Survey 1999–2004. Sarcopenia was broadly defined based on low lean mass (LLM; as measured by dual-energy X-ray absorptiometry), and was defined using appendicular lean mass (ALM) divided by height squared (ALM/height^2^), weight (ALM/weight), and body mass index (BMI; ALM/BMI), respectively. Obesity was defined as BMI ≥ 30 kg/m^2^, body fat percentage ≥ 30/42%, or waist circumference ≥ 102/88 cm. The prevalence of LLM with obesity was estimated according to each ALM index (ALMI). Multivariable Cox regression analysis and sensitivity analysis were used to examine the association between various body composition phenotypes and all-cause mortality.

**Results:**

In older adults with hypertension, the prevalence of LLM with obesity by the ALM/height^2^ index (9.8%) was lower relative to the ALM/weight (11.7%) and ALM/BMI indexes (19.6%). After a median follow-up of 15.4 years, 642 deaths occurred. In the fully adjusted models, LLM with obesity was significantly associated with a higher risk of all-cause mortality (hazard ratio [HR] 1.69, 95% confidence interval [CI] 1.14–2.49, *P* = 0.008; HR 1.48, 95% CI 1.04–2.10, *P* = 0.028; HR 1.30, 95% CI 1.02–1.66, *P* = 0.037; respectively) compared with the normal body phenotype, with no statistical differences found in individuals with LLM or obesity alone. Sensitivity analysis confirmed the robustness of the results.

**Conclusions:**

The prevalence of LLM with obesity markedly differed in older adults with hypertension according to the 3 different ALMIs, varying from 9.8%, 11.7%, to 19.6%. Patients with both LLM and obesity had a higher risk of all-cause mortality. Further large, prospective, cohort studies are warranted to validate these findings and uncover underlying mechanisms.

**Supplementary Information:**

The online version contains supplementary material available at https://doi.org/10.1186/s12877-023-04326-x.

## Introduction

Sarcopenia, a chronic condition denoted by age-related loss of skeletal muscle mass, correlates with declining muscle strength and functional performance, which can result in prolonged recovery period and reduced independence [1, 2]. The prevalence of sarcopenia in older patients (aged ≥ 60 years) ranges from 9.9 to 40.4% according to different diagnostic criteria [3]. This number will certainly increase in countries with aging societies, where sarcopenia has become an increasingly serious medical and public health issue. Accumulating evidence has demonstrated a significant correlation of sarcopenia with metabolic disorders, functional decline, and premature death in the general population [4, 5]. It has been reported that sarcopenia can double individual medical costs and the direct costs attributable to low muscle mass in the US are estimated to exceed $18.5 billion per year [6].

Sarcopenic obesity (SO), a distinct condition of both reduced muscle mass and excess adiposity, also occurs and has raised a continuing concern [7]. The estimated prevalence of SO is up to 11% worldwide, although prevalence rates are numerically higher in subgroups of patients who are aged ≥ 75 years, who are hospitalized, or who are North/South Americans [8]. For middle-aged adults, obesity can accelerate the penetration of fat into muscle, lower physical function, and cause disability, thus increasing the risk of all-cause mortality [9–11]. However, considerable debate has existed regarding the health effects of obesity on older adults, in particular on those with SO, and whether excessive weight may be of benefit [12–14].

Studies have shown that obesity (defined by high body mass index [BMI]) is associated with a decreased risk of all-cause mortality among older adults compared with the control group, which is usually called ‘obesity paradox’ [15]. However, the mortality risk appears to be higher in older patients with SO than in those with sarcopenia and without obesity [16–18]. Nearly three out of four older Americans are likely to have hypertension, which remains a leading cause of disability or death worldwide [19, 20]. Importantly, when hypertension is combined with either obesity or sarcopenia, the mortality risk can be even higher [21, 22]. Considering that ‘obesity paradox’ also disappears in patients with concomitant obesity and sarcopenia, the combination of any two of hypertension, obesity, and sarcopenia may have a synergistic effect of increasing mortality risk in older adults. Nevertheless, it remains unclear whether a copresence of obesity and sarcopenia, or obesity or sarcopenia alone, increases the mortality risk in patients with hypertension.

Obesity and insulin resistance are common in patients with hypertension and may contribute to hypertension via multiple potential mechanisms, including activation of the sympathetic nervous system and the renin-angiotensin-aldosterone system, oxidative stress, and endothelial insulin resistance [23]. The increase in free fatty acids induced by obesity can enhance insulin resistance and then inhibit autophagy by activating mammalian target of rapamycin pathway, thereby causing lysosomal degradation of proteins and organelles in muscle [24]. These findings reveal that in patients with hypertension, obesity and insulin resistance may play a vital role in the pathogenesis of sarcopenia and the prevalence of SO may be higher than the general population. However, uncertainty still exists regarding the prevalence of SO in patients with hypertension.

The aims of this study are to investigate the prevalence of SO in older adults with hypertension, and examine the association between various body composition phenotypes and all-cause mortality, with a focus on SO.

## Methods

### Study population

The National Health and Nutrition Examination Survey (NHANES), undertaken by the National Center for Health Statistics (NCHS) at the Centers for Disease Control and Prevention, is a nationally representative health survey of the US noninstitutionalized civilian population [25]. Based on complex, stratified, multistage, probability clusters, NHANES data are consecutively obtained since 1999 and are released every two years. Detailed information concerning the sample design, weighting, and analytic methodology have been described elsewhere [26]. The NCHS Ethics Review Board has approved the NHANES protocol [27]. Written informed consent was acquired from each participant. This study included older adults aged ≥ 60 years with hypertension from 3 cycles of the NHANES cohort (1999–2000, 2001–2002, and 2003–2004) that collected data on body composition with the full spectrum of age. All individuals were prospectively followed up for mortality through December 31, 2019, permitting more than 15 years of observation for survival outcomes. This study was performed following the Strengthening the Reporting of Observational Studies in Epidemiology (STROBE) guideline for reporting cohort studies (Table S1) [28].

**Fig. 1:**
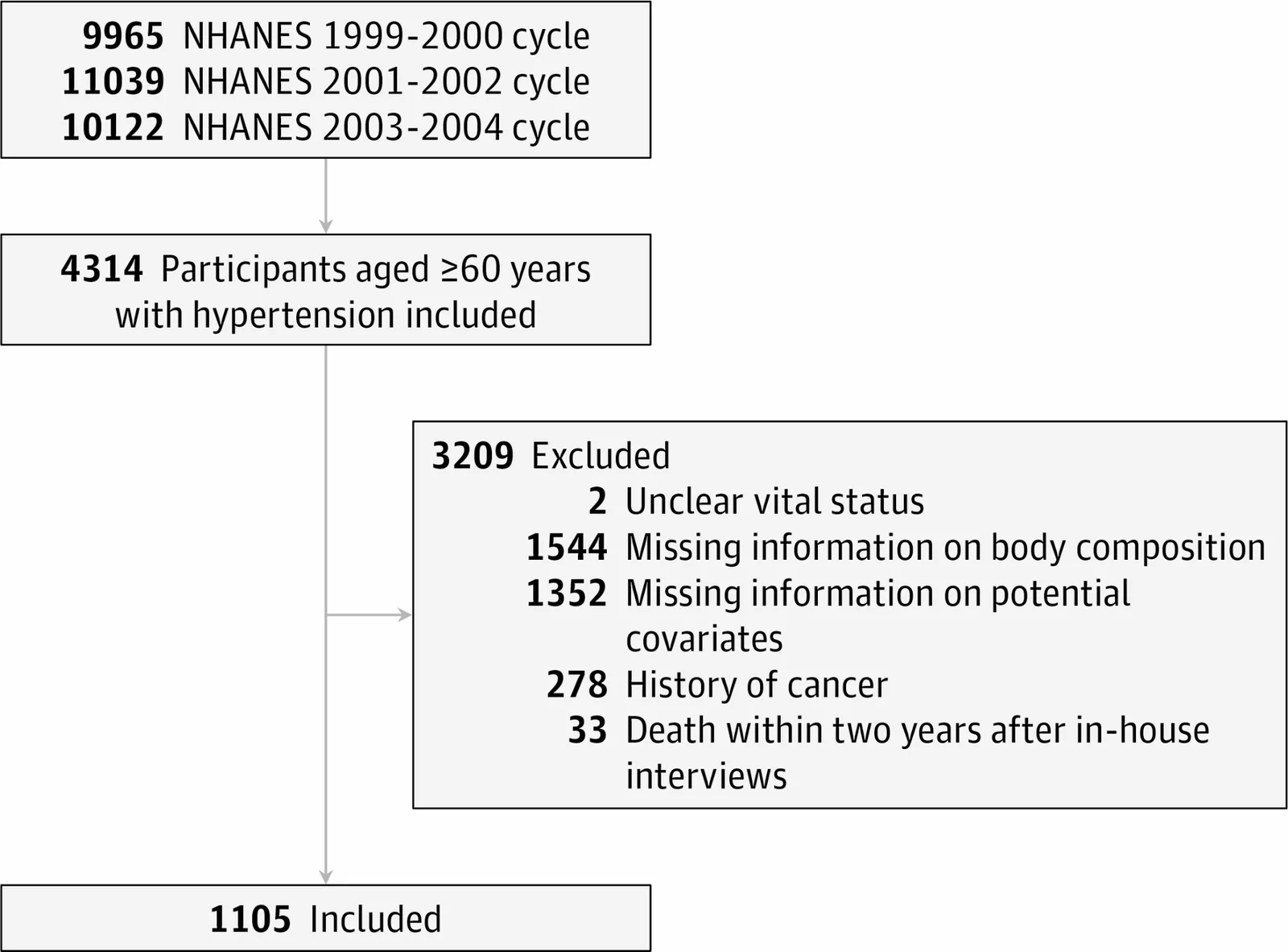
Flow chart of participants included. NHANES, National Health and Nutrition Examination Survey

There were 4314 older adults with hypertension initially identified from the NHANES 1999–2004. Participants with unclear vital status (n = 2) and missing information about body composition or potential covariates (n = 2896) were excluded. Subsequently, we excluded individuals with cancer at baseline (n = 278), because cancer is strongly correlated with the depletion of muscle and fat storage and cachexia may generate large confounding effects. We also excluded individuals dying within the first two years after in-house interviews (n = 33), because those at high risk of all-cause mortality were extremely likely to change their dietary habits and own quite different muscle mass. The final analytical cohort included 1105 individuals (Fig. 1).

### Assessment of hypertension

Following a 5-min rest in a seated position, blood pressure was measured by a certified examiner with a mercury sphygmomanometer according to the recommendations from the American Heart Association [29]. Three or four groups of systolic blood pressure (SBP) and diastolic blood pressure (DBP) were obtained and averaged. Hypertension was defined as (1) mean SBP ≥ 140 mmHg or DBP ≥ 90 mmHg, (2) self-reported physician diagnosis of hypertension, and/or (3) current use of anti-hypertensive medications [30].

### Measurement of body composition

Height, weight, and waist circumference (WC) were measured by a trained examiner following standard procedures and equipment [31]. BMI was calculated as weight divided by height squared (kg/m^2^). WC was measured along the superior lateral border of the iliac crest using a steel tape. Appendicular lean mass (ALM) and body fat (BF) were measured by a certified radiology technologist using whole body dual-energy X-ray absorptiometry (DXA; QDR 4500 A; Hologic, Bedford, MA). ALM was calculated as appendicular lean soft tissue mass excluding body mineral content [32, 33]. Thereafter, ALM was normalized by height squared (ALM/height^2^), weight (ALM/weight), and BMI (ALM/BMI), as suggested by the European Working Group on Sarcopenia in Older People (EWGSOP) [34], Janssen et al. [35], and the Foundation for the National Institutes of Health (FNIH) Sarcopenia Project [36], respectively.

Sarcopenia was broadly defined based on low lean mass (LLM), and was defined using the following 3 sex-specific cutoffs, respectively: (1) ALM/height^2^ < 7.0 kg/m^2^ in males and < 5.5 kg/m^2^ in females; (2) ALM/weight < 0.262 in males and < 0.196 in females; and (3) ALM/BMI < 0.789 in males and < 0.512 in females. Obesity was defined as BMI ≥ 30 kg/m^2^ [37], BF percentage ≥ 30% in males and ≥ 42% in females [38], or WC ≥ 102 cm in males and ≥ 88 cm in females [39], as previously described [40]. Obesity was diagnosed if at least 1 out of the 3 obesity criteria was fulfilled. Subsequently, individuals were divided into 4 groups that combined muscle and fat status (Group 1 no obesity or LLM; Group 2 obesity [without] LLM; Group 3 LLM [without] obesity; and Group 4 LLM with obesity) [41].

### Ascertainment of mortality

Participant outcomes were ascertained via the NHANES Public Use Linked Mortality File, which contains comprehensive information on survival based on the result of a probabilistic match between the NHANES and National Death Index death certificate records through December 31, 2019 [42]. Follow-up duration for each individual was calculated as the time of baseline to the last known date alive or censored from the mortality file.

### Study covariates

To reduce potential confounding from covariates, information on age, sex, race/ethnicity, educational level, family income-to-poverty ratio, smoking status, alcohol intake, physical activity, total protein intake, and chronic health conditions (i.e., diabetes mellitus, chronic kidney disease [CKD], cognitive problem, arthritis, hyperlipidemia, cardiovascular disease, and cancer) was obtained from standardized questionnaires. Race/ethnicity was categorized as non-Hispanic White, non-Hispanic Black, Mexican American, and the other racial/ethnic group. Educational level was classified as less than high school, high school, and college or higher. Family income-to-poverty ratio was divided into < 1.30, 1.30–3.49, and ≥ 3.50, with higher ratios indicating higher levels of household income.

Participants were grouped into nonsmokers, former smokers, and current smokers by the responses about whether they smoked no less than 100 cigarettes during lifetime, and whether they still smoked currently. The mean intakes of alcohol and total protein were obtained from two 24-hour dietary recalls. Participants were divided into nondrinkers (0 g/day), moderate drinkers (0.1–27.9 g/day in males and 0.1–13.9 g/day in females), and heavy drinkers (≥ 28 g/day in males and ≥ 14 g/day in females). Physical activity was measured using metabolic equivalent for the activities relating to frequency and duration of walking/bicycling, tasks around home/yard, and muscle strengthening activities (Table S2) [43]. Physical activity was then categorized as low (< 600 MET-min/week), medium (600–1200 MET-min/week), and high (≥ 1200 MET-min/week) following the World Health Organization guideline recommendations [44].

Diabetes mellitus was defined as (1) self-reported diabetes, (2) hemoglobin A_1c_ levels ≥ 6.5%, (3) fasting glucose levels ≥ 7.0 mmol/L, (4) random glucose levels ≥ 11.1 mmol/L, and/or (5) current use of anti-diabetic medications [45]. Estimated glomerular filtration rate (eGFR) was calculated according to the Chronic Kidney Disease Epidemiology Collaboration equation [46]. A diagnosis of CKD was made if eGFR was less than 60 mL/(min·1.73 m^2^) [47]. Diagnoses of cognitive problem, arthritis, cardiovascular disease, and cancer were determined based on self-reporting using health questionnaires. Hyperlipidemia was defined as (1) triglyceride levels ≥ 150 mg/dL, (2) total cholesterol levels ≥ 200 mg/dL, (3) low-density lipoprotein cholesterol levels ≥ 130 mg/dL, (4) high-density lipoprotein cholesterol levels < 40 mg/dL in males and < 50 mg/dL in females, and/or (5) current use of anti-hyperlipidemic medications [48]. According to the National Cholesterol Education Program’s Adult Treatment Panel III [49], individuals were diagnosed with metabolic syndrome (MetS) when meeting any 3 of the following criteria: elevated WC (≥ 102 cm in males and ≥ 88 cm in females), elevated triglyceride (≥ 150 mg/dL and/or current use of fibrate drugs), low high-density lipoprotein cholesterol (< 40 mg/dL in males and < 50 mg/dL in females and/or current use of niacin drugs), elevated blood pressure (mean SBP ≥ 130 mmHg or DBP ≥ 85 mmHg and/or current use of anti-hypertensive medications), and impaired glucose tolerance (fasting glucose levels ≥ 5.6 mmol/L and/or current use of anti-diabetic medications).

### Statistical analysis

Data analysis was conducted from October 29, 2022 to February 13, 2023. According to the NHANES analytic guideline, primary sampling units, stratification, and sampling weights were used for all statistical analyses to ensure the accurate calculation of the point estimates and standard errors [50]. Based on the 3 criteria for sarcopenia, comparisons of baseline characteristics across various body composition phenotypes (i.e., normal, obesity [without LLM], LLM [without obesity], and LLM with obesity) were performed by one-way analysis of variance for continuous variables and chi-square test for categorical variables.

The prevalence of LLM with obesity, LLM, obesity, and no LLM or obesity in patients with hypertension was calculated. Rates were stratified by the 3 different ALM indexes (ALMIs). Multivariable Cox regression analysis was adopted to calculate the hazard ratios (HRs) and 95% confidence intervals (CIs) to evaluate the association between body composition phenotypes and mortality. Model 1 adjusted for age, sex, and race/ethnicity. Model 2 further adjusted for educational level, family income-to-poverty ratio, smoking status, alcohol intake, physical activity, and total protein intake. Model 3 further adjusted for chronic health conditions. Moreover, we tested the interaction between LLM and obesity with regard to all-cause mortality.

To assess the robustness of the results, two sensitivity analyses were also conducted. First, given that the recently updated clinical practice guidelines recommend the use of 130/80 mmHg as the lower threshold for hypertension, this criterion was chosen as one of the definitions of hypertension in a sensitivity analysis. Second, we investigated whether results from the main analysis were maintained, when obesity was defined using only BF percentage (≥ 30% in males and ≥ 42% in females). All analyses were carried out by R software version 4.1.1 (R Foundation for Statistical Computing, Vienna, Austria). Statistical significance was defined as a two-sided *P*-value < 0.05.

## Results

### Baseline characteristics

Of the 1105 participants, the mean (SE) age was 69.9 (0.2) years, 44.6% of participants were male, and most participants (83.6%) were non-Hispanic White. Notably, ALM normalization methods substantially affected the association between LLM with obesity and MetS; compared with obesity alone, individuals with both LLM and obesity appeared to be at higher risk for MetS by the ALM/weight and ALM/BMI indexes, but not by the ALM/height^2^ index. Baseline demographic and clinical characteristics by the 3 different ALMIs are summarized in Table 1. The LLM group by the ALM/weight index was excluded from further analyses, because no participant was diagnosed with LLM and without obesity. Mean BMI was 28.3 kg/m^2^, mean BF percentage was 31.3% in males and 42.0% in females, and mean WC was 105.2 cm in males and 96.1 cm in females. Elevated WC was the measure that defined the most individuals (69.1%) with obesity, followed by BF percentage (57.2%) and BMI (31.7%). The prevalence of obesity defined by any of BMI, BF percentage, and WC was 76.5% in older adults with hypertension. Table 2 provides detailed information on baseline anthropometry and body composition characteristics.

**Table 1:** Baseline demographic and clinical characteristics of the study population according to the status of obesity and LLM defined by different ALM indexes

Characteristics	Method A (ALM/height^2^)					Method B (ALM/weight)					Method C (ALM/BMI)				
	Normal (n = 177)	Obesity (n = 731)	LLM (n = 97)	LLM with obesity (n = 100)	*P*-value	Normal (n = 274)	Obesity (n = 702)	LLM (n = 0)	LLM with obesity (n = 129)	*P*-value	Normal (n = 248)	Obesity (n = 560)	LLM (n = 26)	LLM with obesity (n = 271)	*P*-value
Age, mean, years	69.2 (0.8)	69.4 (0.3)	72.4 (0.9)	72.6 (0.8)	< 0.001	70.4 (0.6)	69.6 (0.3)	N/A	70.5 (0.6)	0.357	70.3 (0.7)	69.2 (0.3)	71.7 (1.7)	71.5 (0.5)	0.004
Sex					< 0.001					< 0.001					0.002
Female	39.8 (3.8)	56.0 (1.8)	67.7 (5.9)	64.5 (5.6)		50.1 (3.2)	61.9 (1.8)	N/A	30.2 (5.7)		52.2 (3.3)	60.4 (1.8)	22.1 (11.0)	47.4 (4.1)	
Male	60.2 (3.8)	44.0 (1.8)	32.3 (5.9)	35.5 (5.6)		49.9 (3.2)	38.1 (1.8)	N/A	69.8 (5.7)		47.8 (3.3)	39.6 (1.8)	77.9 (11.0)	52.6 (4.1)	
Race/ethnicity					0.010					0.156					< 0.001
Non-Hispanic White	82.8 (2.7)	83.7 (1.9)	82.4 (5.1)	85.4 (4.8)		82.6 (2.7)	82.8 (2.0)	N/A	89.9 (2.7)		84.2 (2.5)	84.0 (2.1)	62.0 (13.3)	83.5 (3.1)	
Non-Hispanic Black	10.9 (2.1)	7.0 (1.1)	2.9 (1.3)	0.4 (0.4)		7.9 (1.4)	6.9 (1.1)	N/A	2.0 (0.7)		8.4 (1.5)	7.8 (1.3)	2.5 (2.5)	1.5 (0.7)	
Mexican American	2.1 (0.6)	3.2 (0.7)	2.3 (0.8)	3.3 (1.2)		2.2 (0.6)	3.2 (0.8)	N/A	3.4 (1.0)		1.9 (0.6)	2.1 (0.5)	6.8 (3.2)	6.6 (1.7)	
Other^a^	4.2 (1.8)	6.1 (1.5)	12.4 (4.5)	10.9 (4.1)		7.2 (2.2)	7.1 (1.6)	N/A	4.7 (2.3)		5.6 (1.9)	6.1 (1.6)	28.7 (12.8)	8.4 (2.4)	
Educational level					0.193					0.154					0.007
Less than high school	11.2 (2.6)	12.3 (1.7)	14.1 (3.8)	11.2 (3.9)		12.3 (2.2)	12.1 (1.8)	N/A	12.4 (3.1)		12.0 (2.3)	10.1 (1.7)	16.7 (7.6)	17.9 (3.3)	
High school	33.8 (5.1)	46.2 (2.2)	40.6 (5.8)	52.5 (7.0)		36.3 (3.9)	46.8 (2.4)	N/A	48.5 (5.7)		36.6 (3.8)	45.3 (2.6)	32.1 (13.5)	52.0 (3.9)	
College or higher	54.9 (5.2)	41.5 (2.4)	45.3 (6.5)	36.3 (5.7)		51.4 (3.9)	41.2 (2.4)	N/A	39.1 (5.5)		51.4 (3.8)	44.5 (2.7)	51.3 (16.1)	30.1 (3.5)	
Family income-to-poverty ratio					0.386					0.030					0.103
<1.30	16.2 (4.2)	19.1 (1.8)	21.0 (5.6)	15.2 (5.1)		18.0 (3.5)	18.6 (1.9)	N/A	19.1 (3.2)		17.8 (3.7)	16.6 (1.9)	19.7 (9.7)	24.7 (3.7)	
1.30–3.49	36.6 (4.8)	44.5 (2.6)	38.1 (5.6)	51.4 (5.1)		37.2 (3.1)	43.7 (2.5)	N/A	54.4 (4.7)		37.1 (3.4)	44.9 (2.7)	38.5 (16.5)	46.8 (3.7)	
≥3.50	47.2 (6.3)	36.4 (2.7)	40.9 (7.3)	33.4 (4.8)		44.9 (4.0)	37.7 (2.9)	N/A	26.4 (3.5)		45.1 (4.7)	38.6 (3.0)	41.8 (16.3)	28.5 (3.8)	
Smoking status					0.242					0.344					0.690
Never	46.1 (4.9)	47.8 (2.3)	56.0 (6.0)	46.4 (5.5)		49.7 (4.3)	49.0 (2.6)	N/A	40.2 (5.5)		50.1 (4.6)	47.3 (2.6)	44.2 (17.0)	48.7 (4.5)	
Former	38.2 (4.7)	43.7 (2.4)	36.5 (6.5)	40.4 (6.5)		37.6 (4.4)	42.3 (2.9)	N/A	48.3 (5.6)		36.8 (4.6)	43.9 (2.9)	48.0 (14.6)	41.3 (4.9)	
Current	15.7 (3.9)	8.5 (1.3)	7.5 (2.7)	13.2 (4.2)		12.7 (2.8)	8.7 (1.3)	N/A	11.5 (3.4)		13.0 (3.1)	8.8 (1.5)	7.7 (4.9)	10.0 (2.2)	
Alcohol intake^b^					0.616					0.801					0.083
Never	79.0 (4.3)	74.5 (1.9)	75.3 (5.4)	66.4 (5.9)		77.6 (3.7)	73.1 (1.9)	N/A	75.6 (4.1)		78.5 (3.9)	70.8 (2.5)	65.3 (11.1)	81.2 (3.0)	
Moderate	13.8 (3.8)	14.0 (1.5)	12.3 (3.8)	20.2 (4.6)		13.2 (3.0)	15.2 (1.7)	N/A	12.7 (3.4)		12.2 (3.1)	15.1 (2.0)	27.3 (9.9)	13.8 (2.7)	
Heavy	7.2 (2.6)	11.5 (1.8)	12.4 (4.1)	13.4 (4.5)		9.1 (2.4)	11.7 (1.8)	N/A	11.8 (3.4)		9.3 (2.5)	14.0 (2.2)	7.4 (7.2)	5.0 (1.6)	
Physical activity, MET-min/week					0.336					0.664					0.423
Low	51.5 (4.8)	57.6 (2.1)	63.2 (5.9)	66.9 (5.8)		55.8 (4.3)	58.4 (2.1)	N/A	61.3 (5.8)		53.9 (4.6)	57.9 (2.6)	81.6 (10.6)	61.6 (3.6)	
Medium	21.7 (3.1)	18.3 (1.9)	13.2 (3.8)	17.2 (5.0)		18.6 (2.6)	17.8 (1.9)	N/A	20.4 (5.0)		19.7 (2.6)	18.3 (2.4)	3.4 (2.2)	17.8 (2.5)	
High	26.8 (3.6)	24.1 (1.9)	23.6 (5.2)	15.8 (4.5)		25.6 (3.1)	23.9 (1.8)	N/A	18.3 (3.1)		26.4 (3.5)	23.9 (2.1)	15.1 (10.3)	20.6 (3.7)	
Total protein intake, mean, g/day	76.8 (3.0)	69.8 (1.3)	68.5 (3.0)	60.9 (2.8)	0.004	73.7 (2.4)	68.8 (1.4)	N/A	68.2 (2.9)	0.214	73.6 (2.7)	70.2 (1.6)	75.8 (5.1)	64.3 (2.1)	0.017
Diabetes mellitus					< 0.001					< 0.001					< 0.001
No	90.3 (2.7)	75.8 (1.7)	87.4 (3.1)	86.5 (4.3)		89.2 (2.0)	78.8 (1.5)	N/A	68.1 (5.4)		88.7 (2.2)	78.2 (1.8)	96.1 (2.8)	74.2 (3.3)	
Yes	9.7 (2.7)	24.2 (1.7)	12.6 (3.1)	13.5 (4.3)		10.8 (2.0)	21.2 (1.5)	N/A	31.9 (5.4)		11.3 (2.2)	21.8 (1.8)	3.9 (2.8)	25.8 (3.3)	
Chronic kidney disease					0.141					0.163					0.044
No	72.3 (3.6)	78.8 (1.7)	70.8 (5.6)	69.8 (6.2)		71.7 (3.0)	78.2 (1.8)	N/A	74.8 (4.3)		72.0 (3.2)	80.3 (1.9)	68.7 (12.1)	70.0 (4.5)	
Yes	27.7 (3.6)	21.2 (1.7)	29.2 (5.6)	30.2 (6.2)		28.3 (3.0)	21.8 (1.8)	N/A	25.2 (4.3)		28.0 (3.2)	19.7 (1.9)	31.3 (12.1)	30.0 (4.5)	
Cognitive problem					0.001					0.030					< 0.001
No	94.8 (1.6)	93.3 (1.0)	83.5 (4.9)	82.7 (5.1)		90.7 (2.3)	93.3 (1.2)	N/A	84.3 (4.3)		90.5 (2.4)	95.0 (1.2)	93.1 (5.0)	83.1 (3.2)	
Yes	5.2 (1.6)	6.7 (1.0)	16.5 (4.9)	17.3 (5.1)		9.3 (2.3)	6.7 (1.2)	N/A	15.7 (4.3)		9.5 (2.4)	5.0 (1.2)	6.9 (5.0)	16.9 (3.2)	
Arthritis					0.404					0.364					0.533
No	59.4 (4.8)	51.8 (2.4)	54.6 (5.8)	56.8 (5.4)		57.6 (3.4)	52.0 (2.7)	N/A	54.8 (4.7)		57.4 (3.4)	52.2 (2.8)	61.1 (9.9)	53.1 (4.2)	
Yes	40.6 (4.8)	48.2 (2.4)	45.4 (5.8)	43.2 (5.4)		42.4 (3.4)	48.0 (2.7)	N/A	45.2 (4.7)		42.6 (3.4)	47.8 (2.8)	38.9 (9.9)	46.9 (4.2)	
Hyperlipidemia					0.051					0.049					0.017
No	16.9 (3.0)	9.1 (1.4)	15.8 (4.0)	16.0 (4.4)		16.5 (2.4)	10.0 (1.4)	N/A	9.7 (2.6)		17.3 (2.4)	10.4 (1.5)	5.6 (3.6)	8.8 (2.0)	
Yes	83.1 (3.0)	90.9 (1.4)	84.2 (4.0)	84.0 (4.4)		83.5 (2.4)	90.0 (1.4)	N/A	90.3 (2.6)		82.7 (2.4)	89.6 (1.5)	94.4 (3.6)	91.2 (2.0)	
Metabolic syndrome					< 0.001					< 0.001					< 0.001
No	82.8 (4.1)	39.5 (2.2)	84.9 (3.9)	63.5 (4.9)		83.6 (3.1)	43.6 (2.3)	N/A	36.9 (5.0)		84.7 (3.2)	43.4 (2.5)	67.9 (12.3)	40.2 (4.6)	
Yes	17.2 (4.1)	60.5 (2.2)	15.1 (3.9)	36.5 (4.9)		16.4 (3.1)	56.4 (2.3)	N/A	63.1 (5.0)		15.3 (3.2)	56.6 (2.5)	32.1 (12.3)	59.8 (4.6)	
Cardiovascular disease					0.795					0.060					0.090
No	69.4 (3.7)	73.5 (1.7)	72.3 (5.7)	73.5 (5.8)		70.4 (3.5)	75.1 (1.6)	N/A	64.8 (5.1)		70.7 (3.6)	75.8 (1.9)	67.6 (10.8)	66.7 (3.9)	
Yes	30.6 (3.7)	26.5 (1.7)	27.7 (5.7)	26.5 (5.8)		29.6 (3.5)	24.9 (1.6)	N/A	35.2 (5.1)		29.3 (3.6)	24.2 (1.9)	32.4 (10.8)	33.3 (3.9)	

Abbreviations: ALM, appendicular lean mass; BMI, body mass index; LLM, low lean mass; MET, metabolic equivalent

Data are presented as weighted mean (SE) for continuous variables or % (SE) for categorical variables

^a^ Other includes other Hispanic, and other race (including multiracial)

^b^ Nondrinker: 0 g/d; moderate drinker: 0.1–27.9 g/d in males and 0.1–13.9 g/d in females; and heavy drinker: ≥28 g/d in males and ≥ 14 g/d in females

**Table 2:** Baseline anthropometry and body composition characteristics of the study population according to the status of obesity and LLM defined by different ALM indexes

Characteristics	Method A (ALM/height^2^)					Method B (ALM/weight)					Method C (ALM/BMI)				
	Normal (n = 177)	Obesity (n = 731)	LLM (n = 97)	LLM with obesity (n = 100)	*P*-value	Normal (n = 274)	Obesity (n = 702)	LLM (n = 0)	LLM with obesity (n = 129)	*P*-value	Normal (n = 248)	Obesity (n = 560)	LLM (n = 26)	LLM with obesity (n = 271)	*P*-value
Height, mean, cm	168.4 (1.0)	166.4 (0.4)	162.3 (1.1)	164.0 (1.1)	< 0.001	166.2 (0.8)	165.7 (0.4)	N/A	168.2 (1.1)	0.063	166.8 (0.8)	167.9 (0.4)	158.1 (0.9)	160.9 (0.7)	< 0.001
Weight, mean, kg	70.1 (1.0)	84.9 (0.6)	55.5 (1.1)	67.1 (1.2)	< 0.001	64.7 (1.1)	81.5 (0.5)	N/A	89.0 (1.8)	< 0.001	65.3 (1.0)	83.8 (0.6)	57.5 (1.7)	79.3 (1.4)	< 0.001
BMI, mean, kg/m^2^	24.6 (0.2)	30.6 (0.2)	21.0 (0.2)	24.9 (0.2)	< 0.001	23.2 (0.2)	29.6 (0.2)	N/A	31.3 (0.4)	< 0.001	23.3 (0.2)	29.6 (0.2)	23.0 (0.6)	30.5 (0.4)	< 0.001
Proportion with BMI ≥ 30 kg/m^2^					< 0.001					< 0.001					< 0.001
No	100.0 (0.0)	52.5 (2.2)	100.0 (0.0)	100.0 (0.0)		100.0 (0.0)	61.8 (2.1)	N/A	40.9 (3.9)		100.0 (0.0)	61.0 (1.9)	100.0 (0.0)	51.6 (4.7)	
Yes	0.0 (0.0)	47.5 (2.2)	0.0 (0.0)	0.0 (0.0)		0.0 (0.0)	38.2 (2.1)	N/A	59.1 (3.9)		0.0 (0.0)	39.0 (1.9)	0.0 (0.0)	48.4 (4.7)	
BF percentage, mean, %	29.8 (0.5)	39.1 (0.3)	32.3 (0.7)	39.9 (0.7)	< 0.001	30.7 (0.4)	39.0 (0.3)	N/A	40.3 (0.7)	< 0.001	30.7 (0.5)	38.8 (0.3)	30.0 (1.5)	40.4 (0.5)	< 0.001
Proportion with BF percentage ≥ 30/42%					< 0.001					< 0.001					< 0.001
No	100.0 (0.0)	25.8 (2.2)	100.0 (0.0)	20.6 (4.7)		100.0 (0.0)	29.5 (2.3)	N/A	1.0 (1.0)		100.0 (0.0)	32.4 (2.5)	100.0 (0.0)	4.2 (1.4)	
Yes	0.0 (0.0)	74.2 (2.2)	0.0 (0.0)	79.4 (4.7)		0.0 (0.0)	70.5 (2.3)	N/A	99.0 (1.0)		0.0 (0.0)	67.6 (2.5)	0.0 (0.0)	95.8 (1.4)	
WC, mean, cm	89.1 (0.6)	105.9 (0.4)	80.7 (0.9)	94.9 (1.1)	< 0.001	86.0 (0.6)	103.3 (0.4)	N/A	111.0 (1.3)	< 0.001	86.1 (0.6)	104.3 (0.5)	85.1 (2.0)	105.0 (1.0)	< 0.001
Proportion with WC ≥ 102/88 cm					< 0.001					< 0.001					< 0.001
No	100.0 (0.0)	6.4 (1.2)	100.0 (0.0)	32.1 (5.2)		100.0 (0.0)	10.8 (1.7)	N/A	3.5 (1.5)		100.0 (0.0)	8.2 (1.7)	100.0 (0.0)	14.0 (2.6)	
Yes	0.0 (0.0)	93.6 (1.2)	0.0 (0.0)	67.9 (5.2)		0.0 (0.0)	89.2 (1.7)	N/A	96.5 (1.5)		0.0 (0.0)	91.8 (1.7))	0.0 (0.0)	86.0 (2.6)	
ALM, mean, kg	20.6 (0.4)	21.2 (0.2)	14.6 (0.4)	15.3 (0.4)	< 0.001	18.4 (0.4)	20.4 (0.2)	N/A	20.8 (0.6)	< 0.001	18.6 (0.4)	21.1 (0.2)	15.8 (0.9)	18.6 (0.4)	< 0.001
ALM/height^2^, mean, kg/m^2^	7.2 (0.1)	7.5 (0.0)	5.5 (0.1)	5.6 (0.1)	< 0.001	6.5 (0.1)	7.3 (0.1)	N/A	7.2 (0.2)	< 0.001	6.6 (0.1)	7.4 (0.0)	6.3 (0.3)	7.1 (0.1)	< 0.001
ALM/weight, mean, %	0.3 (0.0)	0.2 (0.0)	0.3 (0.0)	0.2 (0.0)	< 0.001	0.3 (0.0)	0.2 (0.0)	N/A	0.2 (0.0)	< 0.001	0.3 (0.0)	0.2 (0.0)	0.3 (0.0)	0.2 (0.0)	< 0.001
ALM/BMI, mean, m^2^	0.8 (0.0)	0.7 (0.0)	0.7 (0.0)	0.6 (0.0)	< 0.001	0.8 (0.0)	0.7 (0.0)	N/A	0.7 (0.0)	< 0.001	0.8 (0.0)	0.7 (0.0)	0.7 (0.0)	0.6 (0.0)	< 0.001

Abbreviations: ALM, appendicular lean mass; BF, body fat; BMI, body mass index; LLM, low lean mass; WC, waist circumference

Data are presented as weighted mean (SE) for continuous variables or % (SE) for categorical variables

### Prevalence of LLM with obesity

**Fig. 2:**
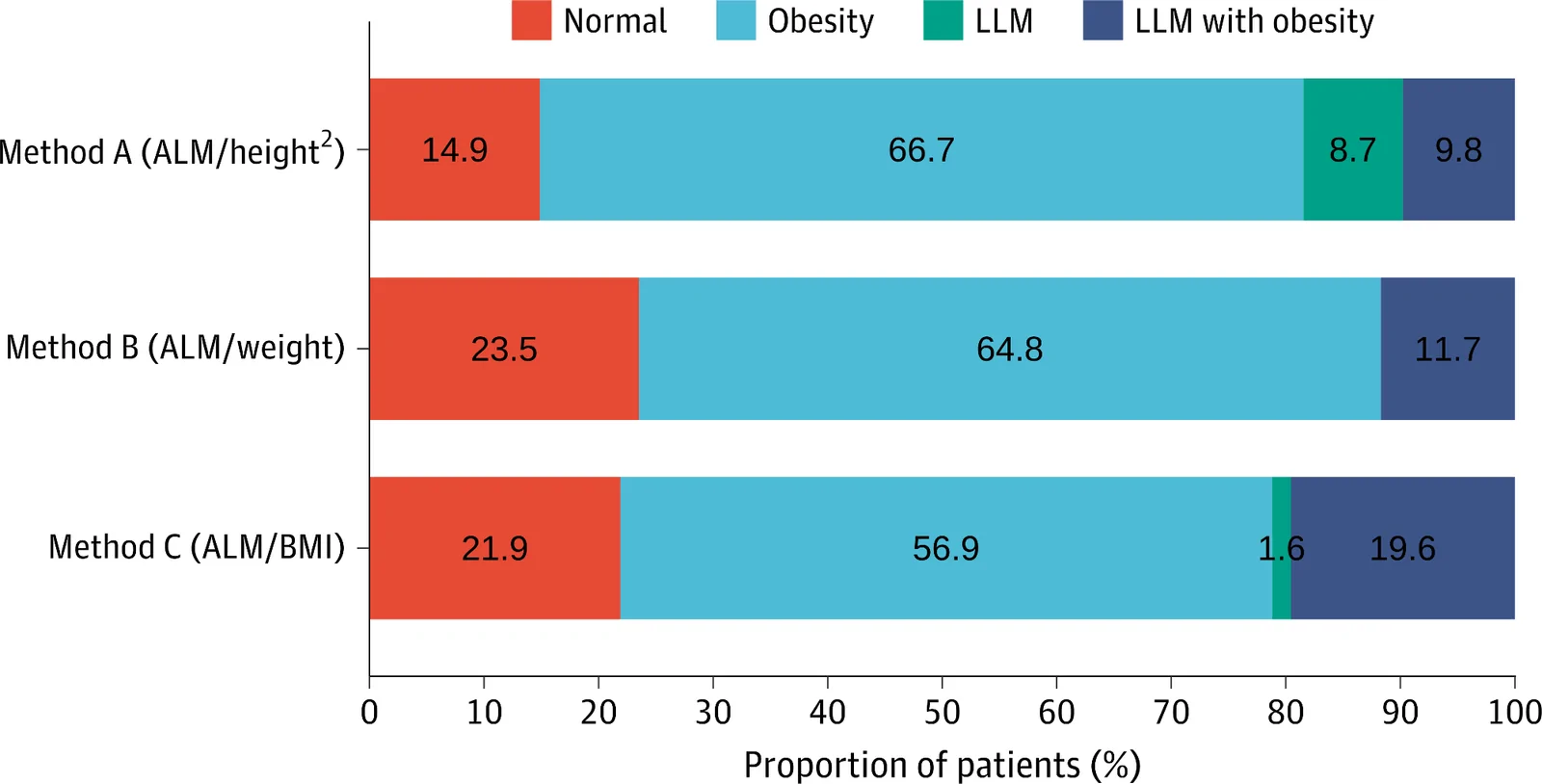
Prevalence of LLM with obesity, LLM, obesity, and no LLM or obesity in patients with hypertension based on different ALM indexes. ALM, appendicular lean mass; BMI, body mass index; LLM, low lean mass

We estimated the prevalence of LLM with obesity, LLM or obesity alone, and normal body phenotype based on 3 different ALMIs (Fig. 2). LLM with obesity was seen in 9.8%, 11.7%, and 19.6% of older adults with hypertension by the ALM/height^2^, ALM/weight, and ALM/BMI indexes, respectively. In relation to LLM alone, prevalence rates ranged from 0% by the ALM/weight index to 8.7% by the ALM/height^2^ index. Additionally, the respective prevalence rates of obesity were 66.7%, 64.8%, and 56.9% using these definitions. Less than one-quarter (14.9%, 23.5%, and 21.9%, respectively) of individuals had neither LLM nor obesity.

### Association between LLM with obesity and all-cause mortality

**Fig. 3:**
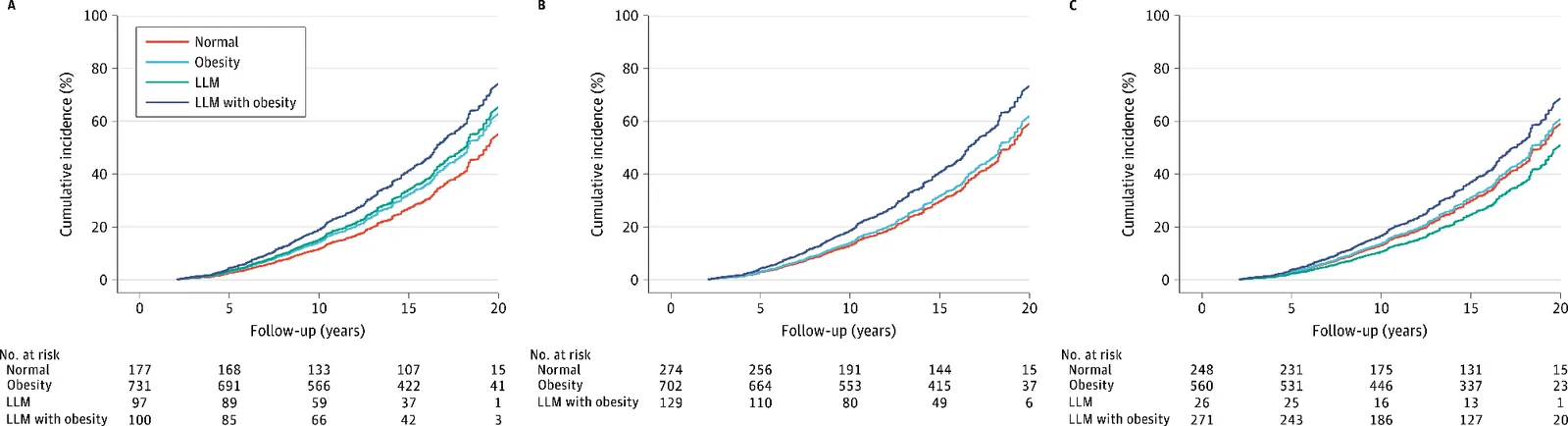
Adjusted cumulative incidence curves of all-cause mortality in patients with hypertension according to the status of obesity and LLM defined by (A) ALM/height^2^, (B) ALM/weight, and (C) ALM/BMI, respectively. ALM, appendicular lean mass; BMI, body mass index; LLM, low lean mass

After 15,148 person-years of follow-up (median duration: 15.4 years; maximum duration: 20.8 years), a total of 642 deaths (56.3%) were observed in older adults with hypertension. Figure 3 illustrates the adjusted cumulative incidence curves of all-cause mortality for various body composition phenotypes according to the ALM/height^2^, ALM/weight, and ALM/BMI indexes, respectively. In the fully adjusted models, LLM with obesity was significantly associated with a higher risk of all-cause mortality (HR 1.69, 95% CI 1.14–2.49, *P* = 0.008; HR 1.48, 95% CI 1.04–2.10, *P* = 0.028; HR 1.30, 95% CI 1.02–1.66, *P* = 0.037; respectively) compared with the normal body phenotype (Table 3). No significant differences were found in individuals with LLM or obesity alone. The adjusted HRs of each body composition phenotype for all-cause mortality were comparable among the 3 different ALMIs. No clinically relevant interaction between LLM and obesity was observed when fitting the fully adjusted model with an interaction term (*P*-values were 0.893 and 0.131 for the ALM/height^2^ and ALM/BMI indexes, respectively).

**Table 3:** HRs and 95% CIs of all-cause mortality in patients with hypertension according to the status of obesity and LLM defined by different ALM indexes

Multivariate models	Method A (ALM/height^2^)				Method B (ALM/weight)				Method C (ALM/BMI)			
	Normal (n = 177)	Obesity (n = 731)	LLM (n = 97)	LLM with obesity (n = 100)	Normal (n = 274)	Obesity (n = 702)	LLM (n = 0)	LLM with obesity (n = 129)	Normal (n = 248)	Obesity (n = 560)	LLM (n = 26)	LLM with obesity (n = 271)
Deaths/person-years	89/2516	408/10,308	72/1158	73/1166	161/3674	384/9947	N/A	97/1527	146/3345	299/8002	15/329	182/3472
Model 1	1.00 (ref.)	1.28 0.99–1.65 *P* = 0.064	1.47 1.08-2.00 *P* = 0.014	1.65 1.09–2.49 *P* = 0.017	1.00 (ref.)	1.05 0.86–1.27 *P* = 0.638	N/A	1.62 1.15–2.27 *P* = 0.005	1.00 (ref.)	1.00 0.80–1.26 *P* = 0.971	0.83 0.50–1.39 *P* = 0.484	1.42 1.11–1.83 *P* = 0.006
Model 2	1.00 (ref.)	1.28 1.01–1.63 *P* = 0.044	1.50 1.04–2.14 *P* = 0.028	1.77 1.22–2.58 *P* = 0.003	1.00 (ref.)	1.05 0.87–1.27 *P* = 0.604	N/A	1.62 1.16–2.26 *P* = 0.004	1.00 (ref.)	1.01 0.82–1.25 *P* = 0.928	0.77 0.36–1.65 *P* = 0.505	1.37 1.09–1.74 *P* = 0.008
Model 3	1.00 (ref.)	1.23 0.95–1.60 *P* = 0.115	1.32 0.91–1.93 *P* = 0.148	1.69 1.14–2.49 *P* = 0.008	1.00 (ref.)	1.08 0.88–1.33 *P* = 0.455	N/A	1.48 1.04–2.10 *P* = 0.028	1.00 (ref.)	1.05 0.83–1.32 *P* = 0.682	0.80 0.41–1.54 *P* = 0.501	1.30 1.02–1.66 *P* = 0.037

Abbreviations: ALM, appendicular lean mass; BMI, body mass index; CI, confidence interval; HR, hazard ratio; LLM, low lean mass

Data are presented as n or weighted HR (95% CI).

Model 1: adjusted for age, sex, and race/ethnicity

Model 2: model 1 + educational level, family income-to-poverty ratio, smoking status, alcohol intake, physical activity, and total protein intake

Model 3: model 2 + diabetes mellitus, chronic kidney disease, cognitive problem, arthritis, hyperlipidemia, and cardiovascular disease

### Sensitivity analysis

When 130/80 mmHg was used as the lower threshold for hypertension, compared with individuals with normal body phenotype, the adjusted HRs (95% CI) of LLM with obesity (1.82 [1.22–2.71], 1.49 [1.07–2.07], and 1.30 [1.03–1.65], respectively) were similar to the cutoff value of 149/90 mmHg, with no significant differences seen in those with LLM or obesity alone (Table S3). Moreover, when we defined obesity exclusively using high BF percentage, LLM with obesity demonstrated a robust trend towards higher risks of mortality versus the normal body phenotype, with HRs (95% CI) of 1.54 (1.04–2.28), 1.47 (1.09–1.99), and 1.32 (1.05–1.66), respectively (Table S4). Results for LLM and obesity remained non-significant after multivariable adjustment, with the exception of the ALM/height^2^ index. When LLM was defined by the ALM/height^2^ index, the risks of all-cause mortality gradually increased from the normal body phenotype to obesity, LLM, and LLM with obesity (*P*_trend_ = 0.014). The prevalence of LLM with obesity in the 2 sensitivity analyses was similar to the main analysis (Figure S1 and Figure S2).

## Discussion

To our knowledge, this study is the first to examine the respective effects of ALM/height^2^, ALM/weight, and ALM/BMI in estimating the prevalence of LLM with obesity in older adults with hypertension, and to reveal the differences in mortality rates between various body composition phenotypes. The main findings are that: (1) the prevalence of LLM with obesity by the 3 different ALMIs differed significantly in older adults with hypertension, varying widely from 9.8%, 11.7%, to 19.6%; and (2) after multivariable adjustment, LLM with obesity was associated with 69%, 48%, and 30% increased risks of all-cause mortality when compared with the normal body phenotype, respectively, with no statistical differences seen in the LLM or obese groups.

There lacks a generally accepted operational definition of SO, which is now considered as a copresence of sarcopenia and obesity [51]; according to different definitions of sarcopenia or obesity, major differences may exist in anthropometric and metabolic characteristics of patients with SO. While sarcopenia has been listed as a disease by the International Classification of Diseases-10 since 2016, its diagnostic criteria are not uniform [52]. Regional expert groups, including the International Working Group on Sarcopenia [53], FNIH Sarcopenia Project [36], EWGSOP2 [34], Asian Working Group for Sarcopenia [54], and Sarcopenia Definition and Outcomes Consortium [55] have formulated a variety of definitions and diagnostic criteria for sarcopenia. Among these definitions, sarcopenia was defined by the absolute or adjusted levels of ALM (i.e., ALM/height^2^, ALM/weight, and ALM/BMI as measured by whole body DXA) to a greater or lesser extent, which was the primary definition used in this study.

Obesity is traditionally defined as BMI ≥ 30 kg/m^2^. However, considerable debate has existed concerning the efficacy of BMI in assessing the association between obesity and mortality in older adults. In a meta-analysis of 32 studies involving 197,940 older adults (aged ≥ 65 years), BMI range of 24.0-29.9 was associated with the lowest risk of mortality, and the mortality risk only began to increase when BMI exceeded ≥ 33.0 kg/m^2^ [56]. Ageing was associated with clinically meaningful changes in body composition, as characterized by a loss of muscle mass and an increase in visceral fat [57]. Therefore, BMI alone may not be a reliable indicator of obesity in older adults, considering that it cannot discriminate between lean mass and fat mass that have opposing effects on mortality [58]. Alternative anthropometric indicators include BF percentage (≥ 30% in males and ≥ 42% in females), WC (≥ 102 cm in males and ≥ 88 cm in females), and waist-hip ratio (≥ 0.90 in males and ≥ 0.85 in females) [59]. Considering the data availability in the NHANES, obesity was defined as any of high BMI, BF percentage, and WC in the main analysis, and was also defined by BF percentage alone in a sensitivity analysis, as previously described [40].

The prevalence of LLM with obesity in this study is hard to compare with other studies, because no related studies in patients with hypertension have been published. In a cross-sectional study in the US elderly population, the prevalence of SO varied up to 26-fold based on the different definitions used [60]. Cauley [61] demonstrated that ALM/height^2^, ALM/weight, and ALM/BMI yielded different distributions of SO after stratification by age, sex, and race/ethnicity; according to different populations and definitions, the prevalence of SO ranged from 0 to 41%. Meng et al. [62] compared the diagnostic efficacy of the ALM/height^2^ and ALM/weight indexes in Chinese older adults aged ≥ 65 years, finding that ALM/weight might be more appropriate in revealing the impact of advanced age on the prevalence of SO. A recent meta-analysis of 167,151 older adults reported the pooled prevalence of SO in the general population [63]. In their study, its prevalence was 8% and 23% based on the ALM/height^2^ and ALM/weight indexes, respectively, which was similar to this study. Evidence suggests that hypertension can facilitate the progression of sarcopenia via stimulating the production of multiple catabolic cytokines [64]. According to the ALM/height^2^ index, the prevalence of sarcopenia was approximately 20.2% in hypertensive patients aged ≥ 60 years [24]; nevertheless, the prevalence of SO is as yet unclear.

This study showed that 9.8%, 11.7%, and 19.6% of older adults with hypertension were diagnosed to have LLM with obesity by the ALM/height^2^, ALM/weight, and ALM/BMI indexes, respectively. Sensitivity analyses confirmed the robustness of the results. Consistent with previous studies, the prevalence of LLM with obesity by the ALM/weight and ALM/BMI indexes was numerically higher in comparison with the ALM/height^2^ index in our study [63]. Because the definitions of obesity were the same for the 3 diagnostic criteria and weight/BMI-based methods for ALM normalization identified rather few patients with LLM (without obesity), obesity defined by weight or BMI might be an independent risk factor for sarcopenia in older adults; these results indicated that ‘obesity paradox’ did not seem to exist in patients with SO. The number/area of intramyocellular lipid droplets and mechanical load of body weight were positively and significantly correlated with myofiber area, which led to greater absolute muscle strength and strength in obese patients [65–67]. Therefore, when normalizing ALM with weight or BMI, the relative muscle mass and strength were weaker, thus resulting in obesity becoming a risk factor for sarcopenia [68]; in contrast, when sarcopenia was defined using the ALM/height^2^ index, older adults with obesity were at a relatively lower risk for sarcopenia.

From another perspective, the ALM/weight and ALM/BMI indexes clearly failed to catch LLM (without obesity), which might be explained by inherent limitations such as anthropometric indicators, statistical methodologies, and diagnostic criteria for sarcopenia and obesity. Specifically, body weight and BMI usually reflect only the sum of BF, muscle, bone, and organs, rather than BF alone [69, 70]. Considering the relatively constant mass of bone and organs, when ALM was adjusted by weight or BMI, individuals with LLM were extremely likely to have a high BF percentage. Hence, the combination of obesity and sarcopenia, as defined by the ALM/weight or ALM/BMI indexes, is of limited benefit in assessing patients with sarcopenia and without obesity. Recently, a third lumbar computed tomography (CT) scan has emerged as a promising development in identifying various body composition phenotypes [71, 72]. CT can distinguish between BF, muscle, bone, water, and air based on tissue-specific attenuation values, thus qualifying total abdominal muscle area, visceral fat area, subcutaneous fat area, and WC directly [73]. Therefore, CT scan, a routine and feasible technique in the clinical setting, may provide a more convenient and accurate diagnostic option for use in older adults with probable obesity, sarcopenia, and SO. Further research is still required to determine the optimal diagnostic criteria for sarcopenia and SO.

Accumulating evidence has demonstrated that sarcopenia was associated with 57%, 75%, and 60% increased risks of hospitalization, rehospitalization, and all-cause mortality, respectively, compared with the non-sarcopenic group [74–76]. Unfortunately, many studies in the field did not discriminate between sarcopenia (without obesity) and SO. It could not be ruled out that a significant fraction of patients with sarcopenia in previous reports actually had SO, which should be regarded as a distinct body composition phenotype with specific clinical and metabolic characteristics. This claim was supported by results from previous studies [40]. Only a few studies have prospectively investigated the association between SO and mortality, and there were indications that older adults with SO had the highest mortality risk compared with other body phenotypes [16–18]. This condition would be further complicated when considering the effect of SO in older adults with hypertension, as hypertension combined with either obesity or sarcopenia might have a higher mortality risk due to the synergistic effect [19, 20]. In this study involving 1105 hypertensive patients, LLM with obesity was significantly correlated with 69%, 48%, and 30% increased risks of all-cause mortality, respectively, as compared with the normal body phenotype; in contrast, no statistical differences were observed in elderly hypertensive patients with LLM or obesity alone. Consistent with our findings, Liu et al. [63] found that the risk of all-cause mortality in patients with obesity alone was comparable with those with normal BMI and without sarcopenia. The ‘obesity paradox’ correlated with mortality might be partly attributed to higher lean soft tissue mass in older adults with obesity alone [77]. For patients with LLM (without obesity), despite including 1105 older adults with hypertension, the sample size was relatively small; only 97, 0, and 26 patients were included according to the 3 ALMIs, respectively, resulting in wide CIs and less precise estimates. Results from several cohort studies showed a significantly higher risk of all-cause mortality in patients with either sarcopenia alone or with SO, with the former contradictory with our results [78, 79]. Therefore, although there was an apparent difference with respect to study population relative to other studies, further large, prospective, longitudinal comparative studies would be beneficial.

## Limitations

This study has several limitations. First, sarcopenia (without obesity) was defined depending solely on muscle quantity, because data on grip strength and physical performance were inaccessible in the NHANES 1999–2004. The use of ALMI alone might not fully capture the complexity of sarcopenia and accurately reflect the true prevalence or influence of sarcopenia in hypertensive individuals. However, different definitions of sarcopenia exist in the current literature, and this definition was commonly recognized and employed in recent studies [80–82]. Second, the findings of this study were primarily applicable to noninstitutional older adults instead of hospitalized patients that were likely to have a greater degree of sarcopenia or obesity. Third, this study was conducted in the US elderly population, presumably limiting the generalizability of the findings to patients from other countries, races/ethnicities, or demographic populations. Fourth, a number of participants (n = 1352) were precluded due to a lack of sufficient clinical data, and thus selection bias could not be completely ruled out. Finally, due to the constraint imposed by the sample size, this study did not investigate the difference in prevalence and mortality rates between men and women; however, a meta-analysis demonstrated no apparent difference in the prevalence of SO between genders in older adults [63]. Future prospective studies in a large cohort are warranted in different ethnic/racial groups.

## Conclusions

The prevalence of LLM with obesity markedly differed in older adults with hypertension according to the ALM/height^2^, ALM/weight, and ALM/BMI indexes, amounting to 9.8%, 11.7%, and 19.6%, respectively. When compared with the normal body phenotype, LLM with obesity was significantly associated with a higher risk of all-cause mortality, with no statistical difference found in patients with LLM or obesity alone. Further large, prospective, cohort studies are required to validate these findings and uncover underlying mechanisms.

## Electronic supplementary material

Below is the link to the electronic supplementary material.

Supplementary Material 1

## Data Availability

The datasets generated and/or analyzed in this study are available at https://wwwn.cdc.gov/nchs/nhanes/default.aspx.

## Abbreviations

ALM: Appendicular lean mass
ALMI: Appendicular lean mass index
BF: Body fat
BMI: Body mass index
CKD: Chronic kidney disease
CT: Computed tomography
DBP: Diastolic blood pressure
eGFR: Estimated glomerular filtration rate
EWGSOP: European Working Group on Sarcopenia in Older People
FNIH: Foundation for the National Institutes of Health
LLM: Low lean mass
MetS: Metabolic syndrome
NHANES: National Health and Nutrition Examination Survey
SBP: Systolic blood pressure
SO: Sarcopenic obesity
WC: Waist circumference
