# Correction: Low lean mass with obesity in older adults with hypertension: prevalence and association with mortality rate

**DOI:** 10.1186/s12877-026-08313-w

**Published:** 2026-09-29

**Authors:** Qiang Qu, Qixin Guo, Jinyu Sun, Xinyi Lu, Lokfai Cheang, Xu Zhu, Wenming Yao, Xinli Li, Haifeng Zhang, Yanli Zhou, Shengen Liao, Rongrong Gao

**Affiliations:** 1 Department of Cardiology, The First Affiliated Hospital of Nanjing Medical University, 300 Guangzhou Road, Nanjing, 210029 China; 2 Department of Cardiology, Suzhou Municipal Hospital, Gusu School, The Affiliated Suzhou Hospital of Nanjing Medical University, Nanjing Medical University, 26 Daoqian Street, Suzhou, 215002 China; 3 Department of Cardiology, Jiangsu Province Hospital, 300 Guangzhou Road, Nanjing, 210029 China

**Correction: BMC Geriatr 23, 619 (2023)**

**https://doi.org/10.1186/s12877-023-04326-x**

Following publication of the original article, the authors would like to correct the information in the first paragraph under the heading Baseline characteristic of the Results section.

The sentence previously read:

Elevated BF percentage was the measure that defined the most individuals (57.2%) with obesity, followed by BMI (31.7%) and WC (28.1%).

The corrected sentence reads:

Elevated WC was the measure that defined the most individuals (69.1%) with obesity, followed by BF percentage (57.2%) and BMI (31.7%).

The original article [1] has been corrected.
