# Supplementary material for: Low lean mass with obesity in older adults with hypertension: prevalence and association with mortality rate
Source: BMC Geriatr. 2023 Oct 3;23:619. doi: 10.1186/s12877-023-04326-x (PMC10546679; doi:10.1186/s12877-023-04326-x)
Supplement: Supplementary file 1 — Supplementary Material 1 [file 12877_2023_4326_MOESM1_ESM.docx]

**Supplemental Material**

**Low lean mass with obesity in older adults with hypertension: prevalence and association with mortality rate**

Qiang Qu, MD, Qixin Guo, MD, Jinyu Sun, MD, Xinyi Lu, MD, Iokfai Cheang, MD, Xu Zhu, MD, Wenming Yao, MD, PhD, Xinli Li, MD, PhD, Haifeng Zhang, MD, PhD, Yanli Zhou, MD, PhD, Shengen Liao, MD, PhD, Rongrong Gao, MD, PhD

[Table S1. Strengthening the Reporting of Observational Studies in Epidemiology (STROBE) reporting guideline for reporting cohort studies checklist 2](#_Toc145786109)

[Table S2. Description of the variables used for measuring physical activity 4](#_Toc145786110)

[Table S3. HRs and 95% CIs of all-cause mortality in patients with hypertension according to the status of obesity and LLM defined by different ALM indexes 5](#_Toc145786111)

[Table S4. HRs and 95% CIs of all-cause mortality in patients with hypertension according to the status of obesity and LLM defined by different ALM indexes 6](#_Toc145786112)

[Figure S1. Prevalence of LLM with obesity, LLM, obesity, and no LLM or obesity in patients with hypertension based on different ALM indexes 7](#_Toc145786113)

[Figure S2. Prevalence of LLM with obesity, LLM, obesity, and no LLM or obesity in patients with hypertension based on different ALM indexes 8](#_Toc145786114)

# **Table S1. Strengthening the Reporting of Observational Studies in Epidemiology (STROBE)** reporting guideline for reporting cohort studies checklist

|  | **Item No** | **Recommendation** | **Page No** |
| --- | --- | --- | --- |
| **Title and abstract** | 1 | (*a*) Indicate the study’s design with a commonly used term in the title or the abstract | 2 |
|  |  | (*b*) Provide in the abstract an informative and balanced summary of what was done and what was found | 2 |
| **Introduction** | | | |
| Background/rationale | 2 | Explain the scientific background and rationale for the investigation being reported | 4 |
| Objectives | 3 | State specific objectives, including any prespecified hypotheses | 5 |
| **Methods** | | | |
| Study design | 4 | Present key elements of study design early in the paper | 6 |
| Setting | 5 | Describe the setting, locations, and relevant dates, including periods of recruitment, exposure, follow-up, and data collection | 6 |
| Participants | 6 | (*a*) Give the eligibility criteria, and the sources and methods of selection of participants. Describe methods of follow-up | 6 |
|  |  | (*b*) For matched studies, give matching criteria and number of exposed and unexposed | N/A |
| Variables | 7 | Clearly define all outcomes, exposures, predictors, potential confounders, and effect modifiers. Give diagnostic criteria, if applicable | 7 |
| Data sources/ measurement | 8^*^ | For each variable of interest, give sources of data and details of methods of assessment (measurement). Describe comparability of assessment methods if there is more than one group | 7 |
| Bias | 9 | Describe any efforts to address potential sources of bias | 10 |
| Study size | 10 | Explain how the study size was arrived at | 6 |
| Quantitative variables | 11 | Explain how quantitative variables were handled in the analyses. If applicable, describe which groupings were chosen and why | 7 |
| Statistical methods | 12 | (*a*) Describe all statistical methods, including those used to control for confounding | 10 |
|  |  | (*b*) Describe any methods used to examine subgroups and interactions | 10 |
|  |  | (*c*) Explain how missing data were addressed | 6 |
|  |  | (*d*) If applicable, explain how loss to follow-up was addressed | 8 |
|  |  | (*e*) Describe any sensitivity analyses | 10 |
| **Results** | | | |
| Participants | 13* | *(a)* Report numbers of individuals at each stage of study—eg numbers potentially eligible, examined for eligibility, confirmed eligible, included in the study, completing follow-up, and analysed | 10 |
|  |  | *(b)* Give reasons for non-participation at each stage | 10 |
|  |  | *(c)* Consider use of a flow diagram | 10 |
| Descriptive data | 14* | *(a)* Give characteristics of study participants (eg demographic, clinical, social) and information on exposures and potential confounders | 10 |
|  |  | *(b)* Indicate number of participants with missing data for each variable of interest | 10 |
|  |  | *(c)* Summarise follow-up time (eg, average and total amount) | 11 |
| Outcome data | 15* | Report numbers of outcome events or summary measures over time | 11 |
| Main results | 16 | (*a*) Give unadjusted estimates and, if applicable, confounder-adjusted estimates and their precision (eg, 95% confidence interval). Make clear which confounders were adjusted for and why they were included | 12 |
|  |  | (*b*) Report category boundaries when continuous variables were categorized | N/A |
|  |  | (*c*) If relevant, consider translating estimates of relative risk into absolute risk for a meaningful time period | 12 |
| Other analyses | 17 | Report other analyses done—eg analyses of subgroups and interactions, and sensitivity analyses | 12 |
| **Discussion** | | | |
| Key results | 18 | Summarise key results with reference to study objectives | 13 |
| Limitations | 19 | Discuss limitations of the study, taking into account sources of potential bias or imprecision. Discuss both direction and magnitude of any potential bias | 17 |
| Interpretation | 20 | Give a cautious overall interpretation of results considering objectives, limitations, multiplicity of analyses, results from similar studies, and other relevant evidence | 15 |
| Generalisability | 21 | Discuss the generalisability (external validity) of the study results | 17 |
| **Other information** | | | |
| Funding | 22 | Give the source of funding and the role of the funders for the present study and, if applicable, for the original study on which the present article is based | 19 |

^*^ Give information separately for exposed and unexposed groups.

# Table S2. Description of the variables used for measuring physical activity^a^

| **Variables** | **Labels** | **Descriptions** | **Answers** |
| --- | --- | --- | --- |
| PAD020 | Walked or bicycled over past 30 days | The next series of questions are about physical activities that {you/SP} {have/has} done over the past 30 days. First, I will ask about activities that are related to transportation. Then, I'll ask about physical activities that {you/he/she} do at school or in {your/his/her} leisure time. Over the past 30 days, {have/has} {you/SP} walked or bicycled as part of getting to and from work, or school, or to do errands? | Yes, no, unable to do activity |
| PAQ050Q | # times walked or bicycled | [Over the past 30 days], how often did {you/SP} do this? [Walk or bicycle as part of getting to and from work, or school, or to do errands.] PROBE: How many times per day, per week, or per month did {you/s/he} do these activities? | Range of values |
| PAQ050U | Unit of measure (day/week/month) | Unit of measure. | Day, week, month |
| PAD080 | How long per day (minutes) | On those days when {you/SP} walked or bicycled, about how long did {you/s/he} spend altogether doing this (minutes)? | Range of values |
| PAQ100 | Tasks around home/yard past 30 days | Over the past 30 days, did {you/SP} do any tasks in or around {your/his/her} home or yard for at least 10 minutes that required moderate or greater physical effort? By moderate physical effort I mean, tasks that caused light sweating or a slight to moder ate increase in {your/his/her} heart rate or breathing. [Such as raking leaves, mowing the lawn or heavy cleaning.] | Code 'unable to do' only if respondent volunteers |
| PAD120 | # of times past 30 days | [Over the past 30 days], how often did {you/SP} do these tasks in or around {your/his/her} home or yard, that is tasks requiring at least moderate effort? [Such as raking leaves, mowing the lawn or heavy cleaning.] PROBE: How many times per day, per week, or per month did {you/s/he} do these activities? | Range of values |
| PAD160 | How long each time (minutes) | About how long did {you/SP} do these tasks each time (minutes)? | Range of values |
| PAD440 | Muscle strengthening activities | Over the past 30 days, did {you/SP} do any physical activities specifically designed to strengthen {your/his/her} muscles such as lifting weights, push-ups or sit-ups? Include all such activities even if you have mentioned them before. | Yes, no, unable to do activity |
| PAD460 | Number of times past 30 days | [Over the past 30 days], how often did {you/SP} do these physical activities? [Activities designed to strengthen {your/his/her} muscles such as lifting weights, push-ups or sit-ups.] | Range of values |

Abbreviations: MET, metabolic equivalent.

^a^ Physical activity was calculated using the following formula: Physical activity in MET-min/week = PAQ050Q (converted to # times per month according to PAQ050U) * PAD080 * 4 / 30 * 7 + PAD120 * PAD160 * 4.5 / 30 * 7 + PAD460 (converted to 30 if ≥31) * 4 / 30 * 7.

# Table S3. HRs and 95% CIs of all-cause mortality in patients with hypertension according to the status of obesity and LLM defined by different ALM indexes^a^

| **Multivariate models** | **Method A (ALM/height^2^)** | | | | **Method B (ALM/weight)** | | | | **Method C (ALM/BMI)** | | | |
| --- | --- | --- | --- | --- | --- | --- | --- | --- | --- | --- | --- | --- |
|  | **Normal**  **(n = 214)** | **Obesity**  **(n = 821)** | **LLM**  **(n = 115)** | **LLM with obesity**  **(n = 108)** | **Normal**  **(n = 328)** | **Obesity**  **(n = 784)** | **LLM**  **(n = 1)** | **LLM with obesity**  **(n = 145)** | **Normal**  **(n = 297)** | **Obesity**  **(n = 625)** | **LLM**  **(n = 32)** | **LLM with obesity**  **(n = 304)** |
| Deaths/person-years | 103/3078 | 434/11800 | 83/1404 | 81/1238 | 186/4465 | 409/11286 | N/A | 106/1751 | 168/4081 | 317/9087 | 18/401 | 198/3951 |
| Model 1 | 1.00 (ref.) | 1.25  0.99-1.57  *P* = 0.061 | 1.43  1.11-1.84  *P* = 0.005 | 1.83  1.22-2.73  *P* = 0.003 | 1.00 (ref.) | 1.04  0.87-1.23  *P* = 0.690 | N/A | 1.72  1.29-2.31  *P* <0.001 | 1.00 (ref.) | 0.98  0.80-1.19  *P* = 0.812 | 0.73  0.42-1.24  *P* = 0.245 | 1.47  1.18-1.83  *P* <0.001 |
| Model 2 | 1.00 (ref.) | 1.24  0.99-1.55  *P* = 0.059 | 1.43  1.07-1.89  *P* = 0.014 | 1.91  1.31-2.78  *P* <0.001 | 1.00 (ref.) | 1.04  0.87-1.24  *P* = 0.656 | N/A | 1.67  1.23-2.26  *P* <0.001 | 1.00 (ref.) | 0.98  0.81-1.18  *P* = 0.814 | 0.65  0.31-1.35  *P* = 0.247 | 1.39  1.11-1.73  *P* = 0.004 |
| Model 3 | 1.00 (ref.) | 1.21  0.94-1.55  *P* = 0.134 | 1.34  0.99-1.82  *P* = 0.061 | 1.82  1.22-2.71  *P* = 0.003 | 1.00 (ref.) | 1.06  0.88-1.27  *P* = 0.555 | N/A | 1.49  1.07-2.07  *P* = 0.019 | 1.00 (ref.) | 1.01  0.82-1.25  *P* = 0.915 | 0.72  0.39-1.35  *P* = 0.311 | 1.30  1.03-1.65  *P* = 0.028 |

Abbreviations: ALM, appendicular lean mass; BMI, body mass index; CI, confidence interval; HR, hazard ratio; LLM, low lean mass.

Data are presented as n or weighted HR (95% CI).

Model 1: adjusted for age, sex, and race/ethnicity.

Model 2: model 1 + educational level, family income-to-poverty ratio, smoking status, alcohol intake, physical activity, and total protein intake.

Model 3: model 2 + diabetes mellitus, chronic kidney disease, cognitive problem, arthritis, hyperlipidemia, and cardiovascular disease.

^a^ Hypertension was defined as (1) mean systolic blood pressure ≥130 mmHg or diastolic blood pressure ≥80 mmHg, (2) self-reported physician diagnosis of hypertension, and/or (3) current use of anti-hypertensive medications.

# Table S4. HRs and 95% CIs of all-cause mortality in patients with hypertension according to the status of obesity and LLM defined by different ALM indexes^a^

| **Multivariate models** | **Method A (ALM/height^2^)** | | | | **Method B (ALM/weight)** | | | | **Method C (ALM/BMI)** | | | |
| --- | --- | --- | --- | --- | --- | --- | --- | --- | --- | --- | --- | --- |
|  | **Normal**  **(n = 358)** | **Obesity**  **(n = 550)** | **LLM**  **(n = 118)** | **LLM with obesity**  **(n = 79)** | **Normal**  **(n = 475)** | **Obesity**  **(n = 501)** | **LLM**  **(n = 1)** | **LLM with obesity**  **(n = 128)** | **Normal**  **(n = 437)** | **Obesity**  **(n = 371)** | **LLM**  **(n = 39)** | **LLM with obesity**  **(n = 258)** |
| Deaths/person-years | 177/5201 | 320/7623 | 86/1413 | 59/911 | 262/6609 | 283/7012 | N/A | 96/1552 | 240/6128 | 205/5220 | 23/486 | 174/3314 |
| Model 1 | 1.00 (ref.) | 1.30  1.07-1.58  *P* = 0.010 | 1.51  1.17-1.96  *P* = 0.002 | 1.45  0.95-2.21  *P* = 0.083 | 1.00 (ref.) | 1.09  0.94-1.26  *P* = 0.244 | N/A | 1.61  1.22-2.14  *P* <0.001 | 1.00 (ref.) | 1.03  0.86-1.24  *P* = 0.758 | 0.87  0.49-1.56  *P* = 0.643 | 1.46  1.19-1.79  *P* <0.001 |
| Model 2 | 1.00 (ref.) | 1.33  1.11-1.58  *P* = 0.002 | 1.55  1.16-2.06  *P* = 0.003 | 1.62  1.08-2.42  *P* = 0.019 | 1.00 (ref.) | 1.13  0.98-1.29  *P* = 0.098 | N/A | 1.64  1.24-2.16  *P* <0.001 | 1.00 (ref.) | 1.08  0.91-1.28  *P* = 0.383 | 0.87  0.44-1.73  *P* = 0.684 | 1.43  1.15-1.76  *P* = 0.001 |
| Model 3 | 1.00 (ref.) | 1.28  1.06-1.55  *P* = 0.012 | 1.39  1.03-1.88  *P* = 0.032 | 1.54  1.04-2.28  *P* = 0.030 | 1.00 (ref.) | 1.14  0.98-1.33  *P* = 0.099 | N/A | 1.47  1.09-1.99  *P* = 0.012 | 1.00 (ref.) | 1.11  0.92-1.34  *P* = 0.286 | 0.90  0.48-1.66  *P* = 0.728 | 1.32  1.05-1.66  *P* = 0.016 |

Abbreviations: ALM, appendicular lean mass; BMI, body mass index; CI, confidence interval; HR, hazard ratio; LLM, low lean mass.

Data are presented as n or weighted HR (95% CI).

Model 1: adjusted for age, sex, and race/ethnicity.

Model 2: model 1 + educational level, family income-to-poverty ratio, smoking status, alcohol intake, physical activity, and total protein intake.

Model 3: model 2 + diabetes mellitus, chronic kidney disease, cognitive problem, arthritis, hyperlipidemia, and cardiovascular disease.

^a^ Obesity was defined as body fat percentage ≥30% in males and ≥42% in females.

# Figure S1. Prevalence of LLM with obesity, LLM, obesity, and no LLM or obesity in patients with hypertension based on different ALM indexes^a^


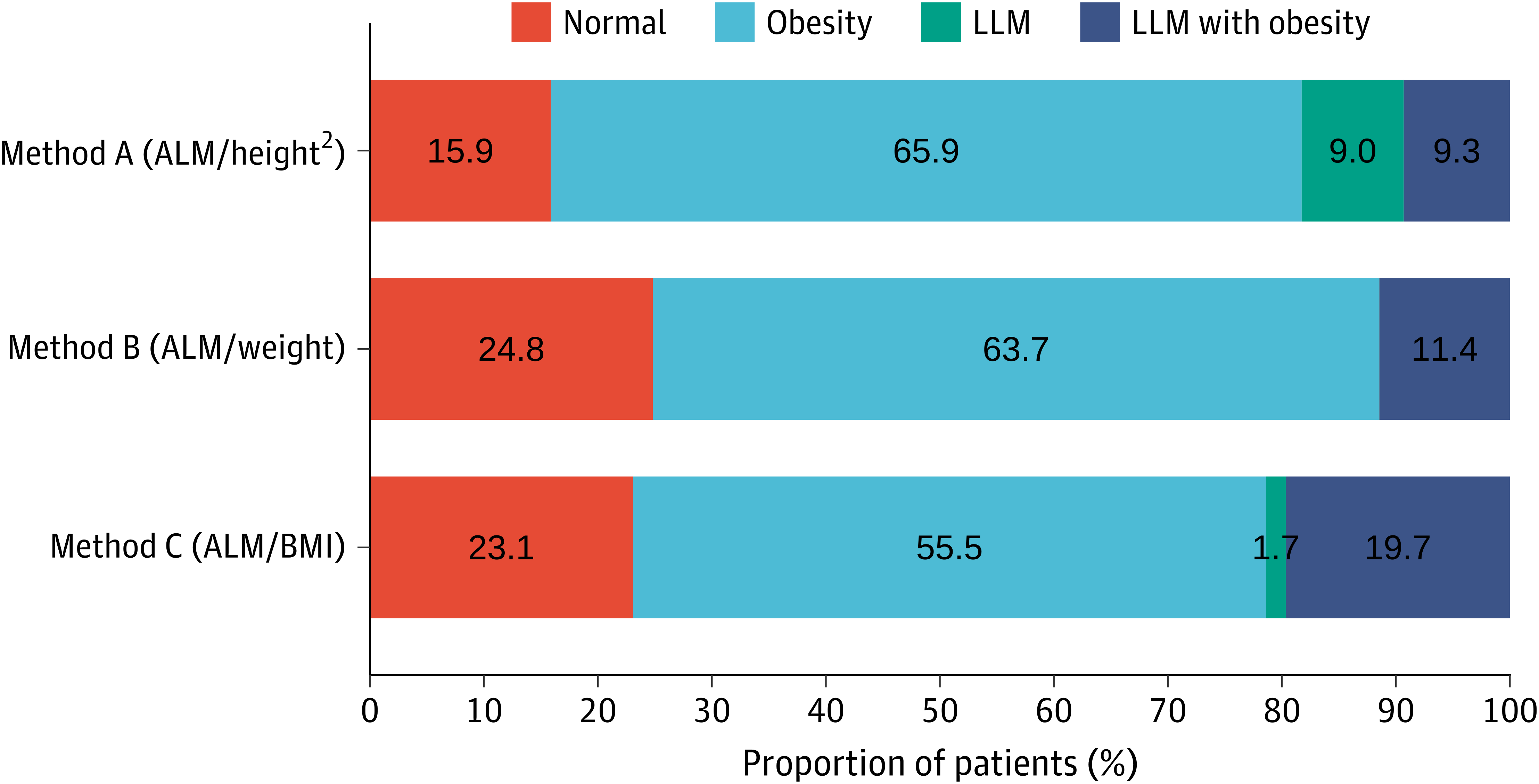


Abbreviations: ALM, appendicular lean mass; BMI, body mass index; LLM, low lean mass.

^a^ Hypertension was defined as (1) mean systolic blood pressure ≥130 mmHg or diastolic blood pressure ≥80 mmHg, (2) self-reported physician diagnosis of hypertension, and/or (3) current use of anti-hypertensive medications.

# Figure S2. Prevalence of LLM with obesity, LLM, obesity, and no LLM or obesity in patients with hypertension based on different ALM indexes^a^


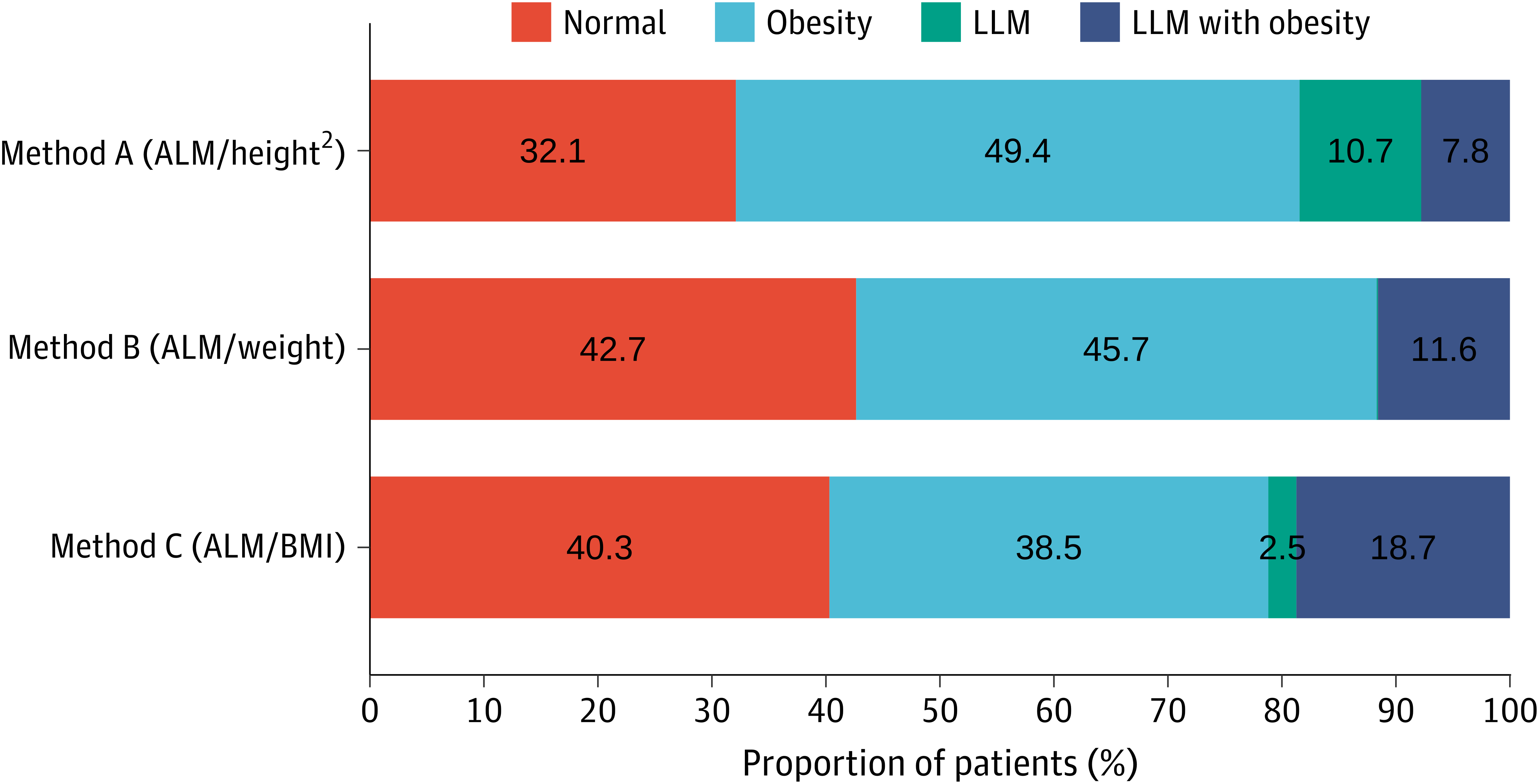


Abbreviations: ALM, appendicular lean mass; BMI, body mass index; LLM, low lean mass.

^a^ Obesity was defined as body fat percentage ≥30% in males and ≥42% in females.
